# The sigma factor σ^54^ (*rpoN*) functions as a global regulator of antibiotic resistance, motility, metabolism, and virulence in *Clostridioides difficile*

**DOI:** 10.3389/fmicb.2025.1569627

**Published:** 2025-04-29

**Authors:** Ying Yang, Tingyu Huang, Junyi Yang, Ruirui Shao, Luhong Shu, Ping Ling, Yingjun Lu, Weihao Ma, Jian Liao, Zhizhong Guan, Guzhen Cui, Xiaolan Qi, Wei Hong

**Affiliations:** ^1^Key Laboratory of Endemic and Ethnic Diseases, Ministry of Education and School/Hospital of Stomatology Guizhou Medical University, Guiyang, China; ^2^Department of Pathology, People’s Hospital of Qiandongnan, Qiandongnan Miao and Dong Autonomous Prefecture, Kaili, Guizhou, China; ^3^Pediatric Intensive Care Unit, Guiyang Maternal and Child Health Care Hospital, Guiyang, China; ^4^Key Laboratory of Microbiology and Parasitology of Education Department of Guizhou, Guizhou Medical University, Guiyang, China; ^5^Collaborative Innovation Center for Prevention and Control of Endemic and Ethnic Regional Diseases Co-constructed by the Province and Ministry, Guiyang, China

**Keywords:** *Clostridioides difficile*, sigma-54, *rpoN*, antibiotic resistance, motility, toxin production, pathogenesis

## Abstract

*Clostridioides difficile*, a major cause of antibiotic-associated diarrhea and pseudomembranous colitis, is increasingly resistant to antibiotics and poses a significant threat due to its regulated virulence. The alternative sigma factor σ^54^ (*rpoN*) is known to regulate gene expression broadly, affecting microbial adaptation. Our study investigates how *rpoN* influences gene expression, physiology, and virulence in *C. difficile*. We used a modified CRISPR-Cpf1 system to create a *rpoN* deletion strain (∆*rpoN*) and a complemented strain (::*rpoN*) in the CD630 background, comparing their phenotypes and transcriptomes with the wild type. The ∆*rpoN* strain showed reduced motility and increased susceptibility to seven antibiotics, including *β*-lactams (amoxicillin, ampicillin, cefoxitin), nitroimidazoles (metronidazole), glycopeptides (vancomycin), fluoroquinolones (norfloxacin), and aminoglycosides (kanamycin). It also exhibited increased toxin gene expression, higher autolysis rates, and enhanced cytotoxicity and virulence in animal models. Additionally, *rpoN* deletion led to a decrease in glucose metabolic rate, which we attribute to the downregulation of glycolytic enzymes. Transcriptomic analysis indicated that reduced motility in ∆*rpoN* is due to downregulation of flagellar biosynthesis genes, while increased autolysis is linked to upregulation of autolysin genes like *cwp19* and *acd*. The enhanced release of toxins due to higher autolysis rates contributes to the increased virulence of ∆*rpoN*. Our findings establish *rpoN* as a global regulator critical for antibiotic resistance, motility, metabolism, toxin production, and pathogenicity in *C. difficile*, suggesting its potential as a therapeutic target to mitigate virulence and resistance.

## 1 Introduction

*Clostridioides difficile* is a spore-forming, strictly anaerobic Gram-positive bacillus and a leading cause of antibiotic-associated diarrhea globally. Severe complications of *C. difficile* infection (CDI)—including pseudomembranous colitis, toxic megacolon, and sepsis—may arise from delayed or inaccurate diagnosis. CDI is further complicated by a 15%–35% recurrence rate, posing significant community and healthcare burdens (Finn et al., 2021). Due to its clinical urgency, the CDC classifies CDI as an immediate public health threat (CDC, 2024).

CDI initiates upon ingestion of environmental spores, which germinate into vegetative cells in the colon (Steindl et al., 2015). Antibiotic use disrupts the gut microbiota, creating a favorable niche for *C. difficile* spore germination into vegetative cells. Toxin A and Toxin B, the primary virulence factors, bind epithelial cell receptors and induce Rho GTPase glucosylation. This disrupts cytoskeletal integrity, triggering cell death and intestinal barrier breakdown. Subsequent influx of luminal contents into the lamina propria manifests as pseudomembranous colitis, characterized by inflammatory patches on the colon mucosa and symptoms such as severe diarrhea, abdominal pain, and fever (Fachi et al., 2024).

In prokaryotes, transcription initiation—a critical regulatory step for gene expression—involves complex mechanisms enabling adaptation to environmental changes (MacDonald et al., 2021). Since the discovery of the sigma (*σ*) factor in 1979, numerous studies have demonstrated its pivotal role in the function of RNA polymerase holoenzyme, influencing the transcription level of various genes (Borukhov and Nudler, 2003). Based on their functions, σ factors can be broadly classified into two categories: the σ^70^ family, which recognizes the −35/−10 region of promoters (Paget, 2015; Sun et al., 2021), and the Sigma-54 (σ^54^) family, which recognizes the −24/−12 region (Yang et al., 2015; Danson et al., 2019). The σ^70^ family, comprising four conserved regions, is the most abundant and primarily regulates gene expression during the exponential growth phase of bacteria (Mazumder and Kapanidis, 2019).

*RpoN* (σ^54^ family, also known as *sigL*) regulates the expression of numerous genes, thereby controlling various bacterial functions (Clark et al., 2022). In *Clostridium acetobutylicum* (Yang et al., 2020), *rpoN* mutants exhibit severely impaired butanol and ethanol production. Overexpression of *rpoN* significantly enhances the production of solvents such as acetone, butanol, and ethanol (Yang et al., 2020). Rukit et al. found that the *rpoN* gene in *Listeria monocytogenes* is involved in bacterial growth and host invasion, and its deletion enhances host invasion (Rukit et al., 2022). In *Pseudomonas aeruginosa*, *rpoN* has been demonstrated as a critical virulence regulator, and deleting the *rpoN* gene reduces its virulence (Lloyd et al., 2017). Additionally, the *rpoN* has been reported to participate in flagellum formation in *Flavobacterium*, thereby regulating the motility of the strain (Yu et al., 2021). Interestingly, studies have revealed that the *rpoN* regulates antibiotic resistance in clinical pathogens such as *Pseudomonas aeruginosa*. Inhibition of *rpoN* expression can increase the susceptibility of strains to antibiotics (Viducic et al., 2016; Lloyd et al., 2019). These findings indicate that *rpoN* is involved in multiple cellular processes and is crucial in regulating bacterial growth and metabolism.

Previous comparative genomic studies of 57 *Clostridium* species, by Nie et al., reconstructed the σ^54^ regulon in *C. difficile* and identified bEBPs and their regulatory modules (Nie et al., 2019). Soutourina et al. employed computational modeling, transcriptomics, and transcription start site mapping to investigate the SigL regulon in *C. difficile* strain 630Δerm, revealing its involvement in the Stickland reaction, a process that generates ATP and NAD^+^ and influences toxin production (Soutourina et al., 2020). Experimental validation confirmed the roles of CdsR and PrdR in cysteine and proline metabolism, respectively (Bouillaut et al., 2013; Gu et al., 2018). Andrew E. Clark’s work underscored the strain-specific nature of SigL’s effects on toxin production, sporulation, and cell surface properties (Clark et al., 2022). While prior studies identified σ^54^-associated regulons in *C. difficile*, strain-specific regulatory roles of RpoN remain unclear. To address this, we generated an *rpoN*-knockout in *C. difficile* CD630 (RT012) using CRISPR-Cpf1. Phenotypic and transcriptomic analyses revealed RpoN’s pleiotropic roles in toxin production, motility, biofilm formation, and virulence, expanding our understanding of its regulatory network.

## 2 Materials and methods

### 2.1 Bacterial strains and culture conditions

All *Escherichia coli* and *Clostridioides difficile* strains used in this study are shown in Supplementary Table S1. NEBExpress Competent *Escherichia coli* cells (C2523H, New England BioLabs) were used for gene cloning and plasmid construction. The CA434 was used as the donor strain for the conjugation transfer of shuttle vectors to *C. difficile* (Purdy et al., 2002). When necessary, chloramphenicol (6 μg/mL), kanamycin (50 μg/mL), and ampicillin (100 μg/mL) were added to the Luria-Bertani (LB) medium for transformants selection. *C. difficile* was grown anaerobically in the brain heart infusion (BHIS) medium supplemented with 5 g/L yeast and 1 g/L L-arginine at 37°C in an anaerobic chamber (AW500TG, Electrotek, United Kingdom). When necessary, streptomycin (15 μg/mL), cephalothin (8 μg/mL), D-cycloserine (250 μg/mL), and 5-fluoroorotic acid (2 mg/mL) were added to the BHIS medium for transformant selection.

### 2.2 Plasmids construction

All plasmids and primers used in this experiment are listed in Supplementary Table S1 (plasmids) and Supplementary Table S2 (primers). Restriction endonucleases were purchased from New England BioLabs (Beijing, China), and DNA polymerase and ClonExpress Multis One Step Cloning were purchased from Novozymes (C115, Nanjing, China).

To enhance the gene-targeting plasmids cure efficiency, we introduced the *pyrF*’ (orotidine-5′-phosphate decarboxylase) gene derived from *Clostridium beijerinckii* NCIMB 8052 in *C. difficile* 630 ∆*pyrF* mutant as a negative selection marker into the pWH34 plasmid backbone (Hong et al., 2018), and then inserted the sRNAP⸬crRNA (*rpoN*) gene-targeting fragment and *rpoN* homologous arms (*rpoN*_arms) to obtain the *rpoN* gene knockout plasmid pYJY3.

The detailed plasmid construction processes were as follows: Firstly, the *pyrF’* gene was amplified with primer pair HW625/HW626 using the genomic DNA of *Clostridium beijerinckii* NCIMB 8052 as the template. The *pyrF’* gene was ligated with *Xho*I-linearized pWH34 to obtain the plasmid pYJY3-S (Supplementary Figure S1B). Subsequently, the sRNAP⸬crRNA (*rpoN*) gene targeting fragment was amplified from pWH37 and simultaneously mutated using the primer pair HW520/HW521. Then, using the *C. difficile* 630 genome as a template, the upstream and downstream homologous arms of the *rpoN* gene were PCR-amplified using the primer pairs HW523/HW524 and HW525/HW526, respectively. Overlapping PCR connected the upstream and downstream homologous arms with the sRNAP⸬crRNA (*rpoN*) targeting fragment. The resulting amplicon was assembled to *Not*I-HF linearized pYJY3-S using the ClonExpress Multis One-Step Cloning kit. The resultant *rpoN* gene knockout plasmid was denoted pYJY3 (Supplementary Figure S1C).

To construct the *rpoN* and *pyrF* gene complementation vector, the *pyrF* and *rpoN* genes with their promoter were amplified from the *C. difficile* 630 genome using the primer pairs HW731/HW734 and HW684/HW685, respectively. The *pyrF* gene fragment was assembled with the *Hind*III-linearized pMTL82151 plasmid to produce pYJY2-S, and the *rpoN* gene fragment was assembled with the *Xho*I-linearized pYJY2-S plasmid to obtain *rpoN* and *pyrF* gene complementation vector pYJY4 (Supplementary Figure S3A).

### 2.3 Construction of ∆*rpoN* and ⸬*rpoN* mutants

Using the heat shock method, the *rpoN* gene knockout plasmid pYJY3 was introduced into the *E. coli* CA434 donor strain (Cañadas et al., 2019). The *E. coli* CA434 strain containing pYJY3 plasmid was used as a donor strain to conjugate the pYJY3 into *C. difficile* 630 ∆*pyrF* mutant (Hong et al., 2018). To screen ∆*rpoN* mutant: (1) The pYJY3-harboring thiamphenicol-resistant colonies (∆*pyrF*) were spread on BHIS solid medium containing 40 mmol/L lactose and 15 μg/mL Tm to screen ∆*rpoN*∆*pyrF*; (2) The deletion of the *rpoN* gene was verified using the primer pair HW557/HW558; (3) Obtained Δ*rpoN*∆*pyrF* mutants were spread on CDMM medium containing 2 mg/mL 5-fluoroorotic acid (5-FOA) to cure the pYJY3 plasmid; (4) The same plasmid conjugation method was used to introduce pYJY2-S and pYJY4 into the Δ*rpoN*∆*pyrF* mutant strain, resulting in complemented strains Δ*rpoN*∆*pyrF*⸬*pyrF* and Δ*rpoN*∆*pyrF*⸬*pyrF*⸬*rpoN.*

### 2.4 Evaluation of gene expression level using RT-qPCR

The WT, Δ*rpoN*, and ⸬*rpoN C. difficile* strains in the logarithmic growth phase were inoculated into fresh BHIS medium and incubated in the anaerobic chamber at 37°C to *OD*_600_ = 0.6. The cells were collected by centrifugation at 13,500×*g* for 3 min. Total RNA was extracted from WT, Δ*rpoN*, and ⸬*rpoN* using a bacterial total RNA extraction kit (DP430, TIANGEN, Beijing). The total RNA was then reverse-transcribed into cDNA using the FasKing gDNA Dispelling RT SuperMix (KR118, TIANGEN, Beijing). The expression levels of the *rpoN* gene in each strain were analyzed using the 16 s ribosomal RNA expression gene (*rrs*) as an internal reference. The amplification primers for *rrs* and *rpoN* genes were HW554/555 and HW693/694, respectively (Supplementary Table S2). The RT-qPCR system was prepared on ice with the 2 × HQ SYBR qPCR Mix (No Rox) kit as follow: 5 μL 2 × HO SYBR gPCR Mix, 0.2 μL Forward Primer (10 mM), 0.2 μL Reverse Primer (10 mM), 1 μL Template cDNA, and 3.6 μL ddH_2_O. The RT-qPCR reaction was performed on the CFX96 Real-Time PCR Detection System (CFX96 Conne, Bio-rad, United States). The data were statistically analyzed using Prism 10 software (Version10.0.3, GraphPad Software, Inc).

To determine the expression levels changes of toxin genes in the WT, Δ*rpoN*, and ⸬*rpoN* strains. These strains in the logarithmic growth phase were each transferred to fresh BHIS medium, with three replicates for each group. The strains were cultivated until they reached an *OD*_600_ of 0.8, after which the cells were collected and RNA was extracted. The 16S ribosomal RNA expression gene (*rrs*) was used as the reference gene. Primers HW554/555, HW887/888, and HW885/886 were used as amplification primers for the *rrs*, *tcdA*, and *tcdB* genes, respectively (Supplementary Table S2). The gene expression levels were detected by RT-qPCR.

### 2.5 Growth profile of strains

The WT, Δ*rpoN*, and ⸬*rpoN* strains were streaked onto solid BHIS agar plates, and single colonies were picked and transferred into BHIS liquid medium. The strains were cultured until they reached the logarithmic growth phase (*OD*_600_ = 0.5), then inoculated into fresh BHIS liquid medium at a 1% inoculation rate and cultured anaerobically at 37°C with three replicates for each group. The *OD*_600_ values were measured every 3 h using a cell density meter (Ultrospec 10, Amersham Biosciences, GE). Growth curves were plotted with time on the x-axis and log_10_(*OD*_600_) values on the y-axis.

### 2.6 Autolysis assay

Overnight cultures of *C. difficile* WT, Δ*rpoN* mutant, and ⸬*rpoN* strains were diluted to *OD*_600_ = 0.05 in BHI and incubated at 37°C until *OD*_600_ = 0.5. Bacterial cells were collected, washed twice, and resuspended in 50 mM potassium phosphate buffer (pH = 7.0), containing 0.01% Triton X-100, to *OD*_600_ = 0.5. The *OD*_600_ of the suspensions were then measured every 20 min at 37°C (*OD*_600_-M), the percent- age of unautolysed cells was calculated as (*OD*_600_-M/0.5)*100%. The untreated cells in the BHIS medium with the same inoculation ratio were set as the control group.

### 2.7 Motility assay

BHIS liquid medium was prepared by adding 0.5% agar to create a semi-solid BHIS medium. The WT, Δ*rpoN*, and ⸬*rpoN* strains in the logarithmic growth phase were streaked on plates and then incubated in an anaerobic chamber at 37°C for 48 h until colonies formed. Single colonies were inoculated into the semi-solid medium in a straight-neck glass tube (diameter 22 mm, specification 10 mL), with triplicates for each group. After cultivation under anaerobic conditions at 37°C for 12 h, the motility results of the strains were recorded.

### 2.8 Hydrogen sulfide (H_2_S) production

The production of H_2_S was determined by the formation of black bismuth sulfide (BS) precipitate through the reaction of bismuth chloride with hydrogen sulfide, with the optical density (*OD*_405_) serving as an indicator (Basic et al., 2015). The WT, Δ*rpoN* and ⸬*rpoN* strains were inoculated in BHIS medium. When the bacterial culture reached *OD*_600_ = 0.6, 100 μL of the bacterial suspension was reacted with an 100 μL of freshly prepared bismuth solution [0.4 M triethanolamine HCl, pH = 8.0; 10 mM bismuth chloride (III), 20 mM 5-phosphor-1-pyrroline, 20 mM EDTA, and 40 mM L-cysteine] were mixed in 96-well plates with 3 replicates per group and reacted in an anaerobic workstation at 37°C for 8 h. During the period, the *OD*_405_ optical density value is measured every 1 h, and the reaction curve is plotted with time as the x-axis and the *OD*_405_ values on the y-axis.

### 2.9 Scanning electron microscopy (SEM)

Single colonies of WT, Δ*rpoN*, and ⸬*rpoN* were picked and cultured in BHIS medium to *OD*_600_ = 0.6, then the bacterial cells were collected by centrifugation at 4,000×*g* for 3 min. The cell pellet was resuspended in a 2.5% glutaraldehyde and fixed overnight at 4°C. The bacterial strains were washed three times with phosphate-buffered saline (PBS) buffer (C10010500BT, Gibco, New York, United States), then dehydrated through graded ethanol of 50, 70, 90, and 100% (vol/vol) for 5 min each. The samples were then dried in a vacuum freeze dryer. After drying, the bacterial powder was carefully picked with a sterilized toothpick. It adhered to a carbon-conductive tape, followed by sputter coating with a gold film in a vacuum evaporator to enhance conductivity and image quality. Finally, a small amount of sample was attached to the tape on a glass slide, and the surface morphology of the strains was observed using a scanning electron microscope (S-3400, Hitachi, Japan).

### 2.10 Antibiotic susceptibility of Δ*rpoN* mutant

The antibiotics resistance of WT, Δ*rpoN*, and ⸬*rpoN* strains to metronidazole, vancomycin, amoxicillin, kanamycin, cefuroxime, norfloxacin, and ampicillin was determined using the series dilution method (Jorgensen and Ferraro, 2009). Each antibiotic was added to the first well of 96-well plate at 128 μg/mL and serially diluted by a factor of two times to create a gradient of antibiotic concentrations: 128, 64, 32, 16, 8, 4, 2, 1, 0.5, and 0.25 μg/mL. Then, 10 μL of WT, Δ*rpoN*, or ⸬*rpoN* strain with an *OD*_600_ of 0.6 was inoculated into each well, with triplicates for each strain. Finally, the cultures were incubated for 24 h at 37°C in an anaerobic chamber, and the *OD*_600_ values were read using a spectrophotometer (Varioskan LUX, Thermo Fisher Scientific, United States). Uninoculated blank controls were set up for all antibiotics. The *OD*_600_ values greater than or equal to 0.1 were considered the growth of *C. difficile* strain.

### 2.11 Single carbon source utilization assay

The WT, Δ*rpoN*, and ⸬*rpoN* strains were cultured to an *OD*_600_ of 0.6 and then inoculated into 5 mL of single carbon source medium (Ehsaan et al., 2016) at a 1% inoculation rate. The cultures were continuously incubated anaerobically at 37°C for 72 h. The *OD*_600_ values were recorded every 3 h during the logarithmic growth phase and every 12 h during the stationary phase, and growth curves were plotted accordingly.

### 2.12 Biofilm assays

The influence of *rpoN* on *C. difficile* biofilm formation was evaluated using a biofilm assay as previously reported (Pantaléon et al., 2015) with mirror modifications. Overnight *C. difficile* cultures were diluted 1: 200 in BHIS broth and distributed into 24-well plates, 2 mL per well. Plates were wrapped to prevent evaporation and incubated anaerobically at 37°C for 48 h. Following incubation, the culture supernatant was removed and then incubated at 37°C with 0.2% (w/v) crystal violet to fix and stain. The crystal violet was subsequently removed, and the biofilms were washed an additional two times with PBS and photographed. To measure biofilm formation, crystal violet retained by the biomass was released with anhydrous ethanol and quantitated by detecting the *OD*_562_ value in a technical quadruplicate for three individual wells.

### 2.13 Determine toxins level using western blot

In order to further elucidate variations in the protein levels of TcdA and TcdB in the different mutants, Western blot analysis was employed to detect the expression levels of TcdA and TcdB in WT, Δ*rpoN*, and ⸬*rpoN* strains. The supernatant was collected by centrifuging 5 mL of *OD*_600_ = 0.6 bacterial culture at 3,440×*g*, followed by sterile filtration through a 0.22 μm sterile filter membrane. The total protein content in the supernatant was determined using the BCA method (P0012S, Beyotime), and 6 μg of protein-containing supernatant was subjected to SDS-PAGE. Subsequently, the proteins in the gel were transferred to a PVDF membrane (IPVH00010, Merck) and subjected to immunoblotting using a 1:1,000 dilution of TcdA and TcdB antibodies (*Clostridioides difficile*, EPR23359-15/EPR23357-19, ab272720/ ab270452, Abcam). The blots were developed using a 1:10,000 dilution of horseradish peroxidase-conjugated rabbit anti-mouse IgG antibody (A0208, Beyotime), with triplicates for each sample.

### 2.14 Cell cytotoxicity assay

Vero cell line were selected for cytotoxicity assay with the WT, Δ*rpoN* and ⸬*rpoN* strains. Vero cells were cultured in DMEM (Dulbecco’s Modified Eagle’s Medium-high glucose) supplemented with 10% fetal bovine serum (Catalog Number, Gibco, United States) and 1% dual antibodies (10 mg/mL streptomycin, 10,000 U/mL penicillin). Vero cells were seeded onto 24-well plates and incubated at 37°C until the wells were confluent. The medium was then removed, and the cells were washed three times with sterile PBS buffer. Subsequently, 200 μL of DMEM (without antibiotics and serum) was added, and the cells were cultured for an additional 24 h. The WT, Δ*rpoN*, and ⸬*rpoN* strains were anaerobically incubated in an anerobic chamber until *OD*_600_ = 1.0, and 1 mL of the bacterial culture was centrifuged at 13,500×*g* for 5 min. The supernatant containing *C. difficile* toxins was filtered through a 0.22 μm sterile filter membrane (QFC06-SF022P25, Zeren Technology Co, Shenzhen, China). The toxin-containing supernatant was diluted from 0.5 × 10^1^ to 0.5 × 10^10^, and 200 μL of each toxin dilution was used to infect the Vero cells. The cells were incubated overnight at 37°C. The cell morphology was observed and photographed under a light microscope at a magnification (CKX53SF, Olympus, Tokyo, Japan) of 200×, and the highest dilution factor that caused cell rounding in Vero cells was determined.

### 2.15 Construction of CDI animal model and the pathology of WT, ∆*rpoN*, and ⸬*rpoN* strains

Syrian golden hamsters (4–5 weeks old, ~100 g) were purchased from Liaoning Changsheng Biotechnology Co., Ltd. and housed 5 per cage with ad libitum access to food and water. Hamsters were acclimated for 1 week prior to the experiment. To disrupt the gut microbiota, hamsters in the experimental groups (WT, Δ*rpoN*, and ⸬*rpoN*) were administered a cocktail of antibiotics (kanamycin 0.8 mg/mL, gentamicin 0.07 mg/mL, polymyxin B 0.1135 mg/mL, metronidazole 0.43 mg/mL, and vancomycin 0.09 mg/mL) in drinking water for 7 days (Chen et al., 2008; Geeraerts et al., 2015; Mooyottu et al., 2017). Control animals received PBS. On day 9, all hamsters were subcutaneously injected with clindamycin (10 mg/kg) to further perturb the gut microbiota (Chen et al., 2008). On days 10, 12, and 14 post-clindamycin injection, hamsters were gavaged with different *Clostridioides difficile* strains or PBS (control). Each hamster received 100 μL of bacterial suspension (*OD*_600_ = 0.6, 4 × 10^5^ CFU/mL). Body weight and fecal consistency were monitored daily.

### 2.16 Transcriptome data processing and analysis

The WT, Δ*rpoN*, and ⸬*rpoN* strains were inoculated into BHIS medium and cultured until the *OD*_600_ reached 0.6. The cultures were then centrifuged at 4,000×*g* for 10 min, the supernatant was removed, and the bacterial pellets of each strain were collected. The samples were transported with adequate dry ice in an foam box to Nanjing Personalomics Co., Ltd. for transcriptome sequencing. Initially, RNA extraction and quality assessment were performed. Once the samples were deemed satisfactory, the following steps were conducted for sequencing: (1) Removal of ribosomal RNA; (2) Enrichment and purification of mRNA; (3) Fragmentation of mRNA; (4) Construction of the sequencing library and quality assessment of the library; (5) Sequencing on the Illumina Novaseq 6,000 platform (Zhou et al., 2024).

Raw data organization, filtering, and quality assessment: after the samples are sequenced, sequence image files are obtained, and the sequencing platform software converts the data into raw data in FASTQ format with a size of 4 GB (Raw Data). The RNA-seq raw data (RNA-seq of *Clostridioides difficile* Δ*rpoN* mutant against wild-type control) was deposited in the ArrayExpress database under the accession number of E-MTAB-14512. The following treatments are applied to the raw data: Initially, data quality control is performed, which includes quality control of base quality distribution and Base Content of the sequenced data; Subsequently, to avoid interference with subsequent information analysis, further filtering is conducted on low-quality reads; Then, the obtained transcriptome data is aligned and analyzed against the reference genome GCF_000009205.2_ASM920v2_genomic.fna (NC_009089.1 and NC_008226.2) on NCBI; Finally, expression quantification analysis is performed on the measured genome. Since read counts are positively correlated with the actual expression levels of genes, gene length, and sequencing depth, to make the gene expression levels comparable between different genes and samples, the expression levels are normalized using FPKM (Fragments Per Kilobase of transcript per million mapped reads), and genes with an FPKM > 1 are considered to be expressed (Radakovits et al., 2012).

Cluster analysis of differentially expressed genes (Tatusov et al., 2003): Cluster analysis is used to determine the expression patterns of differentially expressed genes under different experimental conditions; genes with high expression correlation between samples are categorized into one group, which usually indicates that these genes have actual connections in certain biological processes or metabolic and signaling pathways. Therefore, we can discover unknown biological connections between genes through expression clustering. We use the R language Pheatmap package (version 1.0.12) for two-way hierarchical cluster analysis of the union of differential genes and samples in all comparison groups, clustering based on the expression levels of the same gene in different samples and the expression patterns of different genes in the same sample, using the Euclidean method to calculate distances, and the longest distance method (Complete Linkage) for clustering.

Functional enrichment analysis of differentially expressed genes (McClure et al., 2013): The DEseq software (version 1.44.0) is used to analyze the differentially expressed mRNA, with mRNA considered differentially expressed when the FoldChange (fold change) > 2 and the *p*-value < 0.05. A volcano plot of the differentially expressed mRNA is generated using the R language ggplot2 package (version 3.5.1). The differentially expressed mRNA target genes were subjected to Gene Ontology (GO) and Kyoto Encyclopedia of Genes and Genomes (KEGG) enrichment analysis. All target genes are mapped to each item in the GO database, the number of differentially expressed target genes in each item is calculated, and a hypergeometric distribution is used to calculate the significantly enriched target genes, with *p* ≤ 0.05 considered as significantly enriched pathways.

### 2.17 Statistical methods

Prism 10 (Version 10.0.3) was used for statistical analysis. Two-group comparisons were performed using students’ t-tests or non-parametric tests. Comparisons among multiple groups were performed using one-way ANOVA, while comparisons among multiple groups over time were conducted using two-way ANOVA, followed by Tukey’s multiple comparisons. The results were expressed as mean ± standard deviation, with a test level of *α* = 0.05, and *P* < 0.05 was statistically significant (*n* = 3). *P* > 0.1, *n*; *P < 0.05; **P < 0.01; ***P< 0.001; ****P < 0.0001.

## 3 Results

### 3.1 Phenotypic characterization of mutant strains

**Figure 1:**
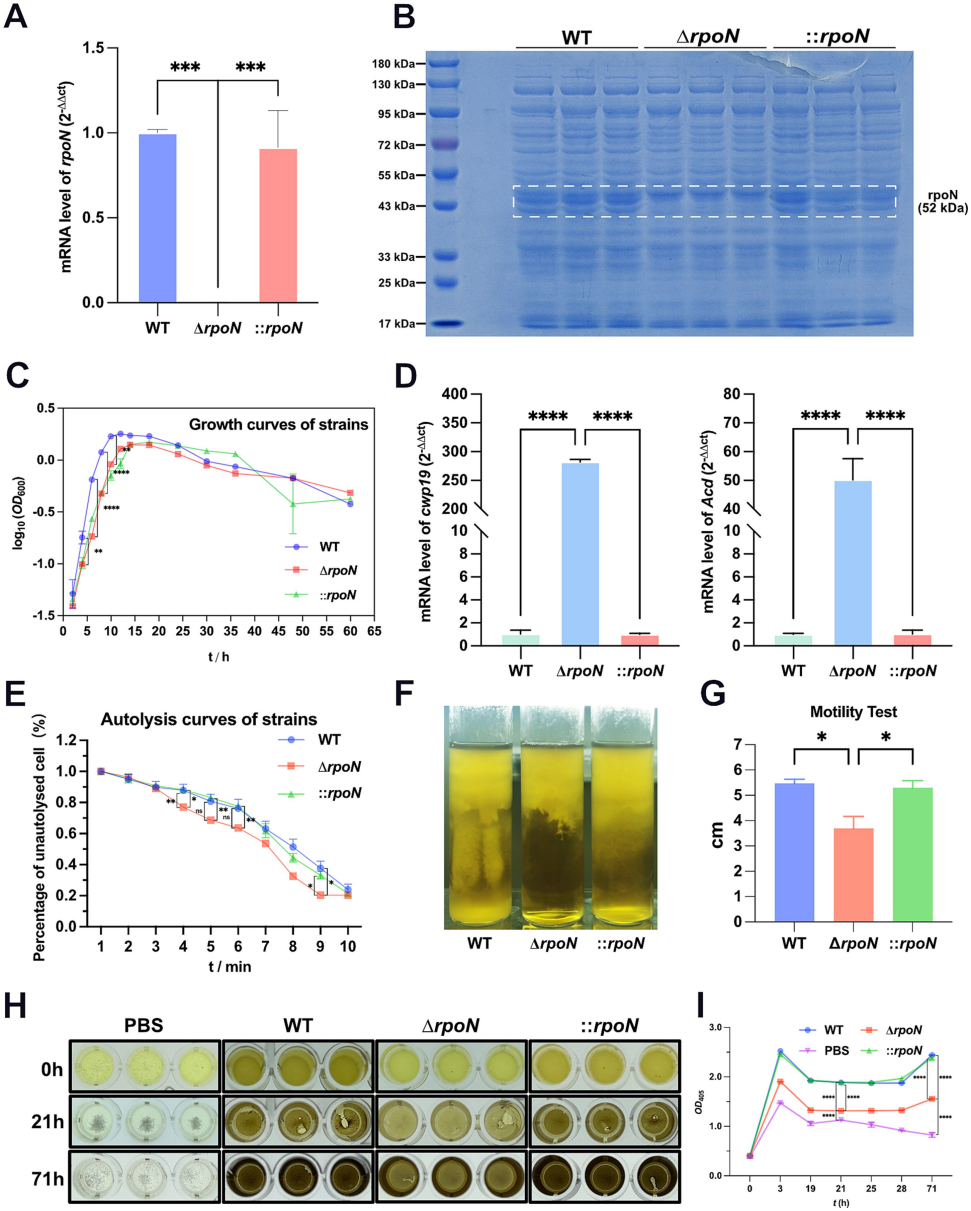
Phenotypic characterization of mutant strains. **(A)** RT-qPCR showed that in the expression of the *rpoN* gene no longer detectable in the ∆*rpoN* mutant, whereas it was restored in the ⸬*rpoN* complementary strain. Data are presented as mean ± SEM. Statistical significance was determined using one-way ANOVA, followed by Tukey’s *post hoc* test. *p ≤ 0.05, **p ≤ 0.01, ***p ≤ 0.001, **** *p* ≤ 0.0001; **(B)** Verification of ∆*rpoN* mutant by using SDS-PAGE. The results showed that the ∆*rpoN* mutant strain absented a 52 kDa protein band compared to the WT strain, and it was restored in the ⸬*rpoN* strain; lane M molecular weight marker (from top to bottom, 180 kDa, 130 kDa, 95 kDa, 72 kDa, 55 kDa, 43 kDa, 33 kDa, 25 kDa, and 17 kDa). **(C)** Growth curves of WT, ∆*rpoN*, and ⸬*rpoN* strains, the horizontal coordinate is incubation time (hours), and the vertical coordinate is cell turbidity at *OD*_600_; **(D)** The green, blue, and red bars indicate the expression levels of the *cwp19* or *Acd* genes in the WT, Δ*rpoN*, and ⸬*rpoN* strains, respectively; **(E)** Cell autolysis rate of the WT, ∆*rpoN*, and ⸬*rpoN* strains, the horizontal coordinate is treatment duration of Triton X-100, the vertical coordinate is the percentage of unautolysed cells; **(F,G)** Comparison of motility of the WT, ∆*rpoN*, and ⸬*rpoN* strains, the motility of the ∆*rpoN* mutant strain was significantly decreased than that of the WT and the ⸬*rpoN* strains; **(H,I)** Comparison of hydrogen sulfide (H_2_S) production between the WT, Δ*rpoN*, and ⸬*rpoN* strains, the Δ*rpoN* mutant strain produced significantly less H_2_S than the WT, while the ⸬*rpoN* strain restored the ability to produce H_2_S. Data are presented as mean ± SEM. Statistical significance was determined using two-way ANOVA, followed by Tukey’s post hoc test in the growth curves, the autolysis curves and the H_2_S production curves. Motility test and the expression of autolysis-related genes were compared using one-way ANOVA. *p ≤ 0.05, **p ≤ 0.01, ***p ≤ 0.001, ****p ≤ 0.0001.

Initially, we verified the successful generation of the Δ*rpoN* [also known as *sigL* (Clark et al., 2022)] mutant strain by confirming its presence at both the genomic DNA and protein levels. RT-qPCR confirmed complete loss of *rpoN* expression in Δ*rpoN*, while complementation (⸬*rpoN*) restored expression to wild-type (WT) levels (Figure 1A). SDS-PAGE revealed the absence of the 52.1 kDa RpoN protein in Δ*rpoN*, which was rescued in ⸬*rpoN* (Figure 1B). The above results suggest that the construction of the *C. difficile* 630 strain Δ*rpoN* and ⸬*rpoN* strains was successful.

To assess the impact of *rpoN* on *C. difficile* 630 phenotype, we investigated changes in growth rate, motility, hydrogen sulfide (H_2_S) production, autolysis rate, and the expression of autolysis-related genes (Meouche and Peltier, 2018; Noori Goodarzi et al., 2022). During the logarithmic growth phase (0–12 h), the Δ*rpoN* strain exhibited a significantly reduced growth rate and maximum biomass compared to the WT strain. While all strains showed a short stationary phase, the Δ*rpoN* strain displayed a significantly higher autolysis rate than the WT. The Δ*rpoN* strain’s growth rate and maximum biomass were similar to those of the ⸬*rpoN* strain, but its autolysis rate was significantly lower than both the WT and Δ*rpoN* strains (Figure 1C).

To investigate the cause of the increased autolysis rate in the Δ*rpoN* strain, we quantified the expression of two known autolysis genes, *cwp19* (Meouche and Peltier, 2018) and *acd*, using RT-qPCR (Noori Goodarzi et al., 2022). Results showed that both genes were significantly upregulated in the Δ*rpoN* strain (Figure 1D). Consistent with this, autolysis assays revealed a significantly higher autolysis rate for the Δ*rpoN* strain compared to the WT and ⸬*rpoN* strains (Figure 1E). Motility assays demonstrated that the Δ*rpoN* strain exhibited significantly reduced motility compared to both the WT and ⸬*rpoN* strains (*p* < 0.05) (Figures 1F,G). Furthermore, H_2_S production was significantly lower in the Δ*rpoN* strain compared to the WT and ⸬*rpoN* strains (Figures 1H,I). In summary, the Δ*rpoN* strain exhibited decreased growth rate, motility, and H_2_S production, coupled with an increased autolysis rate.

### 3.2 Changes in cell surface morphology

**Figure 2:**
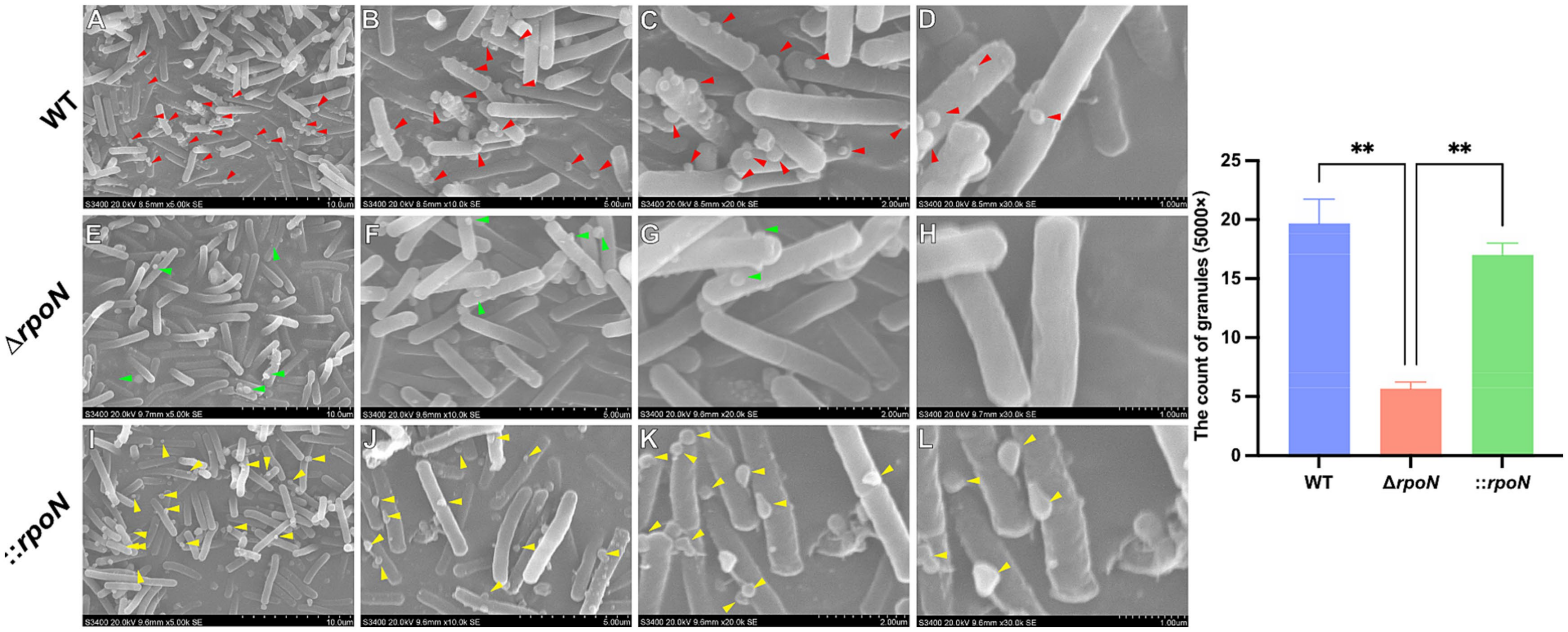
Surface morphology of WT, ∆*rpoN* and ⸬*rpoN* strains viewed by Scanning Electron Microscopy. **(A–D)** Cell surface morphology of the WT strain under ×5,000 **(A)**, ×10,000 **(B)**, ×20,000 **(C)**, and ×30,000 **(D)** magnifications; **(E–H)** Cell surface morphology of ∆*rpoN* strain under ×5,000 **(E)**, ×10,000 **(F)**, ×20,000 **(G)**, and ×30,000 **(H)** magnifications; **(I–L)** Cell surface morphology of ⸬*rpoN* strain under ×5,000 **(I)**, ×10,000 **(J)**, ×20,000 **(K)**, and ×30,000 **(L)** magnifications. The arrows indicate cell surface deposits (granules). The blue, red, and green bars represent the number of granules in WT, Δ*rpoN*, and ⸬*rpoN* strains, respectively (Three fields of view at ×5,000 were selected, and each view was counted three times).

To investigate whether the increased autolysis rate of the ∆*rpoN* strain relates to changes in cell surface structure, we examined the surface morphology of WT, ∆*rpoN*, and ⸬*rpoN* strains using Scanning Electron Microscopy (SEM). Results showed abundant granules (Chu et al., 2016) attached to the surface of the WT strain (Figures 2A–D). In contrast, the ∆*rpoN* strain exhibited a significant reduction in granule attachment (Figures 2E–H). Importantly, the ⸬*rpoN* strain displayed granule attachment levels comparable to those of the WT strain (Figures 2I–L).

### 3.3 Deletion of *rpoN* significantly affects antibiotic resistance of *Clostridioides difficile*

**Figure 3:**
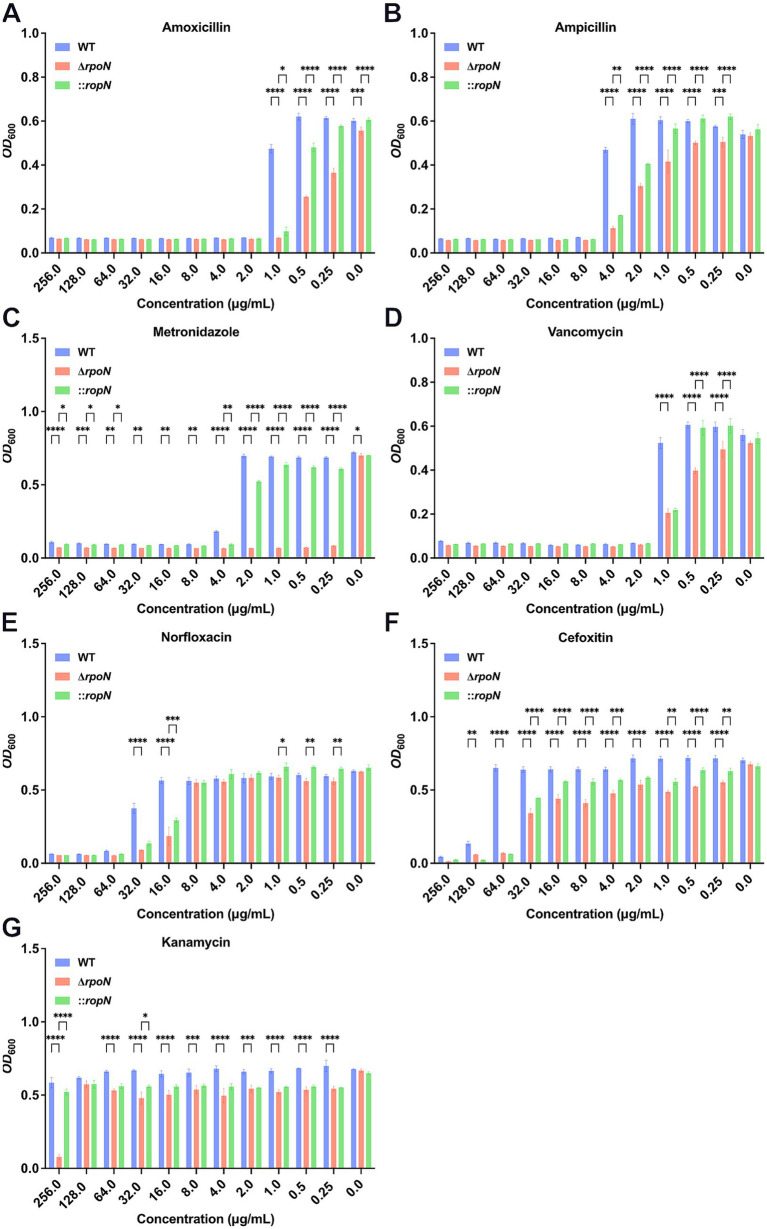
Antibiotic susceptibility of WT, ∆*rpoN*, and ⸬*rpoN* strains. The vertical axis represents the *OD*_600_ value, while the horizontal axis represents the concentration of different antibiotics (mg/mL). The blue, red, and green bars indicate the *OD*_600_ values of WT, Δ*rpoN*, and ⸬*rpoN* strains at different antibiotic concentrations. Compared to WT, the Δ*rpoN* strain exhibited significantly decreased susceptibility to amoxicillin **(A)**, ampicillin **(B)**, metronidazole **(C)**, vancomycin **(D)**, norfloxacin **(E)**, cefotetan **(F)**, and kanamycin **(G)**, whereas the ⸬*rpoN* strain showed partial restoration of susceptibility to these antibiotics compared to WT.

RpoN is an alternative sigma factor involved in transcriptional regulation, microbial physiology, stress resistance, and pathogenicity. To assess the impact of *rpoN* on antibiotic resistance, we compared the resistance profiles of WT, Δ*rpoN*, and ⸬*rpoN* strains to amoxicillin (Figure 3A), ampicillin (Figure 3B), metronidazole (Figure 3C), vancomycin (Figure 3D), norfloxacin (Figure 3E), cefoxitin (Figure 3F), and kanamycin (Figure 3G). The Δ*rpoN* strain exhibited significantly reduced resistance to all tested antibiotics compared to the WT (Figures 3A–G). Resistance profiles of the ⸬*rpoN* strain partially restored to WT levels. These results indicate that the *rpoN* gene plays a significant role in modulating antibiotic resistance in *C. difficile* 630.

### 3.4 Deletion of rpoN upregulates TcdA/TcdB expression and cytotoxicity in *Clostridioides difficile*

**Figure 4:**
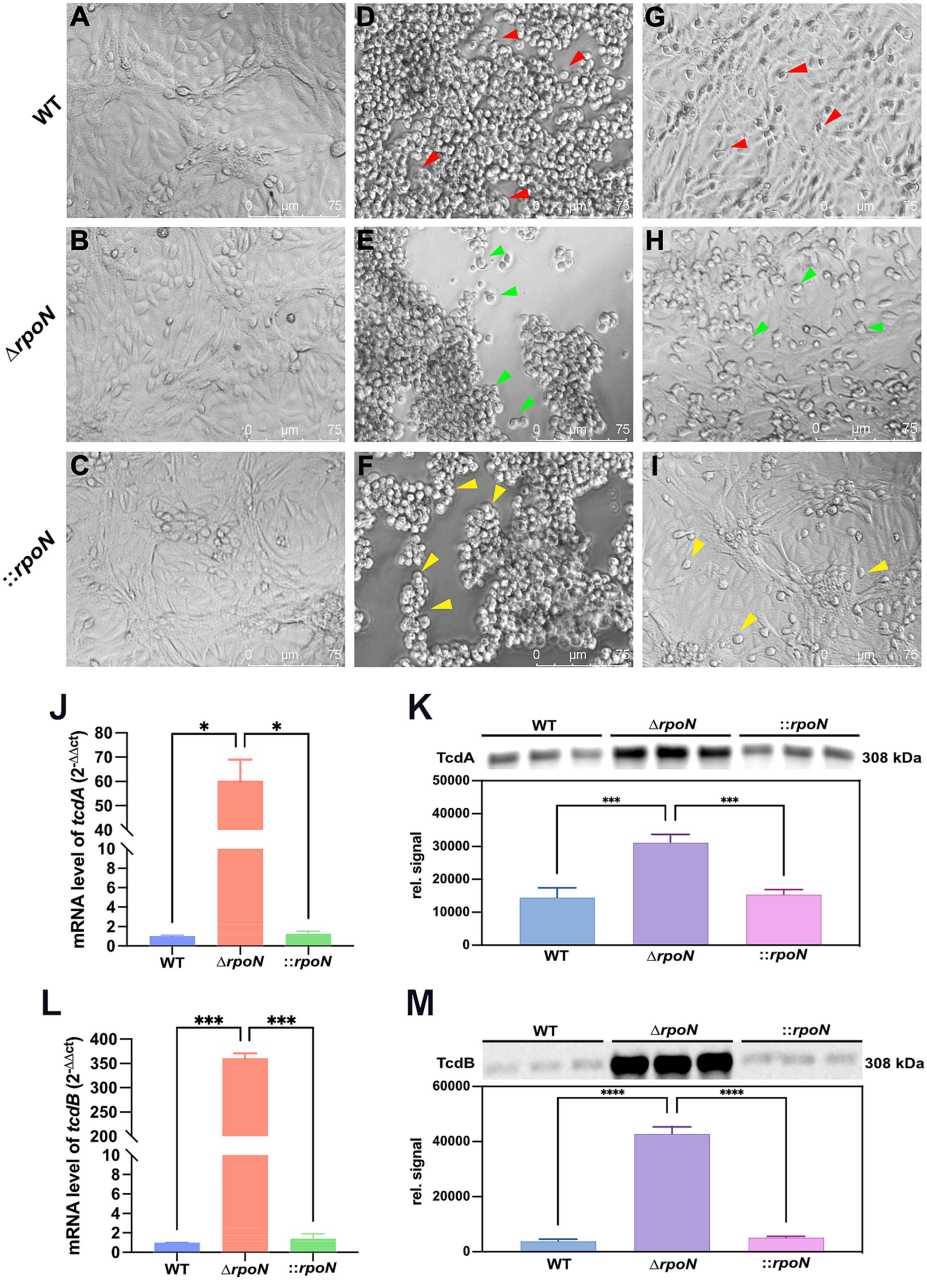
Cell toxicity of WT, ∆*rpoN* and ⸬*rpoN* strains. Supernatants of each strain were co-cultured with Vero cells, and morphological changes of Vero cells were observed under a light microscope. **(A–C)** Show the PBS control without toxins, where cells exhibit a healthy spindle shape. Vero cells cocultured with undiluted WT **(D)**, ∆*rpoN* **(E)**, and ⸬*rpoN* **(F)** supernatants became rounded and suspended (red, green, and yellow arrows); **(G)** Shows the results of Vero cells co-cultured with WT supernatant toxin diluted 0.5 × 10^7^ times; **(H)** Shows the results of Vero cells co-cultured with ∆*rpoN* supernatant toxin diluted 0.5 × 10^9^ times; **(I)** Shows the results of Vero cells co-cultured with ⸬*rpoN* supernatant toxin diluted 0.5 × 10^8^ times; **(J,L)** Blue, red, and green bars represent the expression levels of tcdA or tcdB genes in WT, ∆*rpoN*, and ⸬*rpoN* strains, respectively; **(K,M)** Blue, purple, and pink bars represent the expression levels of TcdA or TcdB proteins in WT, ∆*rpoN*, and ⸬*rpoN* strains (detected by western blot), respectively.

To further investigate the impact of *rpoN* on cytotoxicity, we assessed the virulence of WT, Δ*rpoN*, and ⸬*rpoN* strains using a Vero cell model. Vero cells were infected with graded dilutions (0.5 × 10^1^ to 0.5 × 10^10^) of supernatant from each strain. Control cells (no supernatant) exhibited normal fusiform morphology with clear cell junctions (Figures 4A–C). Infection with undiluted supernatants from WT (Figure 4D), Δ*rpoN* (Figure 4E), and ⸬*rpoN* (Figure 4F) resulted in rounded, suspended cells with disrupted cell junctions. The highest supernatant dilutions that induced morphological changes in Vero cells were 0.5 × 10^7^ (Figure 4G), 0.5 × 10^9^ (Figure 4H), and 0.5 × 10^8^ (Figure 4I) for WT, Δ*rpoN*, and ⸬*rpoN*, respectively. Consequently, the Δ*rpoN* strain exhibited a 100-fold increase in cytotoxicity compared to the WT, while the ⸬*rpoN* strain displayed intermediate cytotoxicity.

To determine the mechanism underlying the increased cytotoxicity of the Δ*rpoN* strain, we examined the expression levels of *tcdA* and *tcdB*. RT-qPCR analysis revealed significantly increased mRNA expression of both toxin genes (Figures 4J,L). Western blot analysis confirmed a corresponding increase in TcdA and TcdB protein levels (Figures 4K,M). These findings indicate that deletion of *rpoN* leads to increased expression of TcdA and TcdB, resulting in enhanced cytotoxicity. This suggests that RpoN may act as a repressor of toxin gene expression in *C. difficile*.

### 3.5 The Δ*rpoN* mutant exhibits reduced biofilm formation

**Figure 5:**
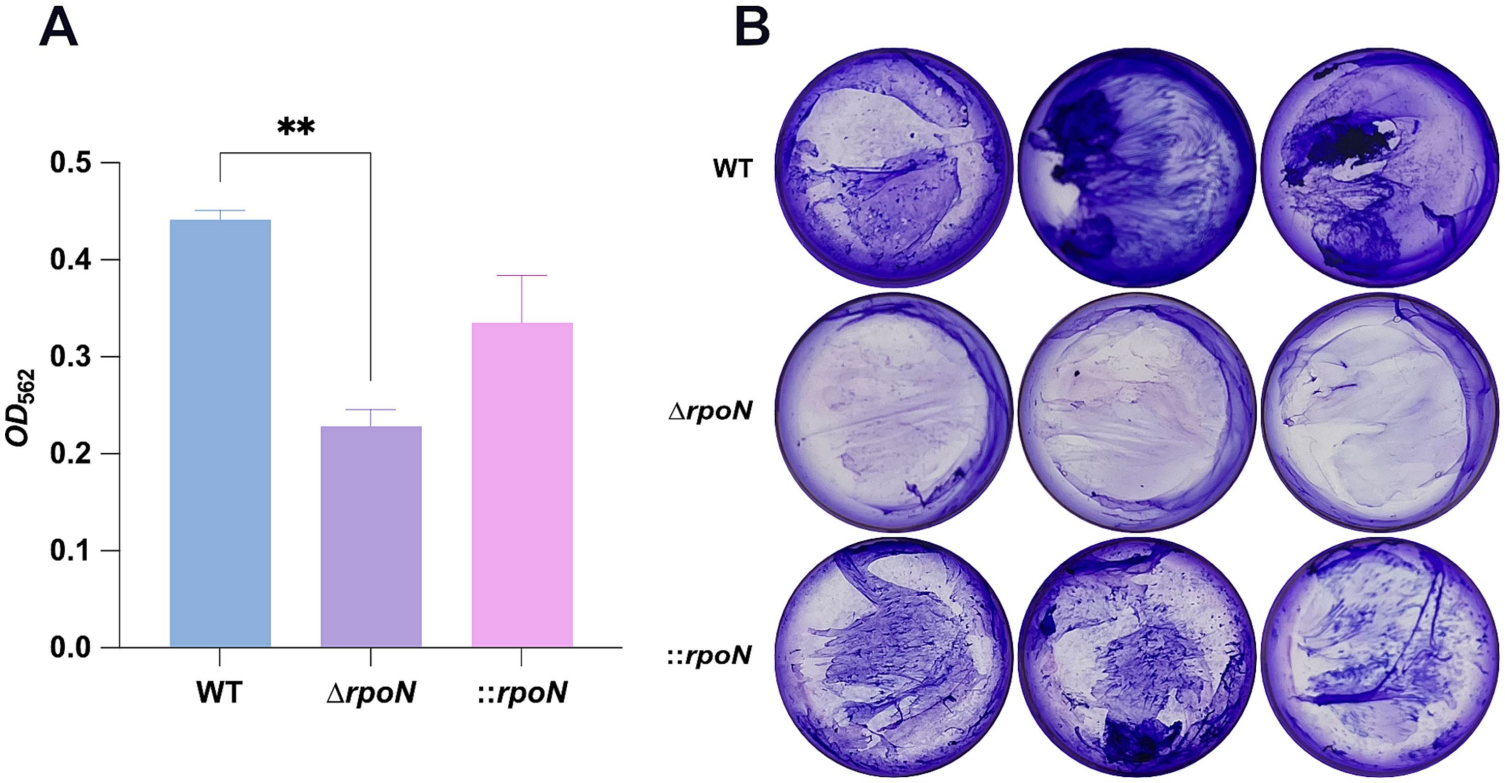
Biofilm assay for WT, ∆*rpoN* and ⸬*rpoN* strains. **(A)** Biofilm formation in WT, ∆*rpoN* and ⸬*rpoN* strains, which was assessed by measuring the retention of crystal violet by *C. difficile* biomass after 48 h; **(B)** Blue, purple, and pink bars represent the *OD*_562_ value of the WT, ∆*rpoN* and ⸬*rpoN* strains, which represent biofilm formation capability. The formed biofilm is reduced in ∆*rpoN* strain. Samples were grown in biological triplicate and compared using one-way ANOVA. *p ≤ 0.05, **p ≤ 0.01, ***p ≤ 0.001, ****p ≤ 0.0001.

Biofilm formation contributes to *C. difficile* persistence in the gut and influences pathogenesis (Pantaléon et al., 2015; Chamarande et al., 2021). Screening for biofilm formation using a 24-well plate assay revealed that the Δ*rpoN* mutant formed less dense biofilms compared to the WT strain. Complementation of the mutant partially restored biofilm formation (Figures 5A,B).

### 3.6 Comparative transcriptomic analyses of the WT and Δ*rpoN* mutant gene expression profiles

**Figure 6:**
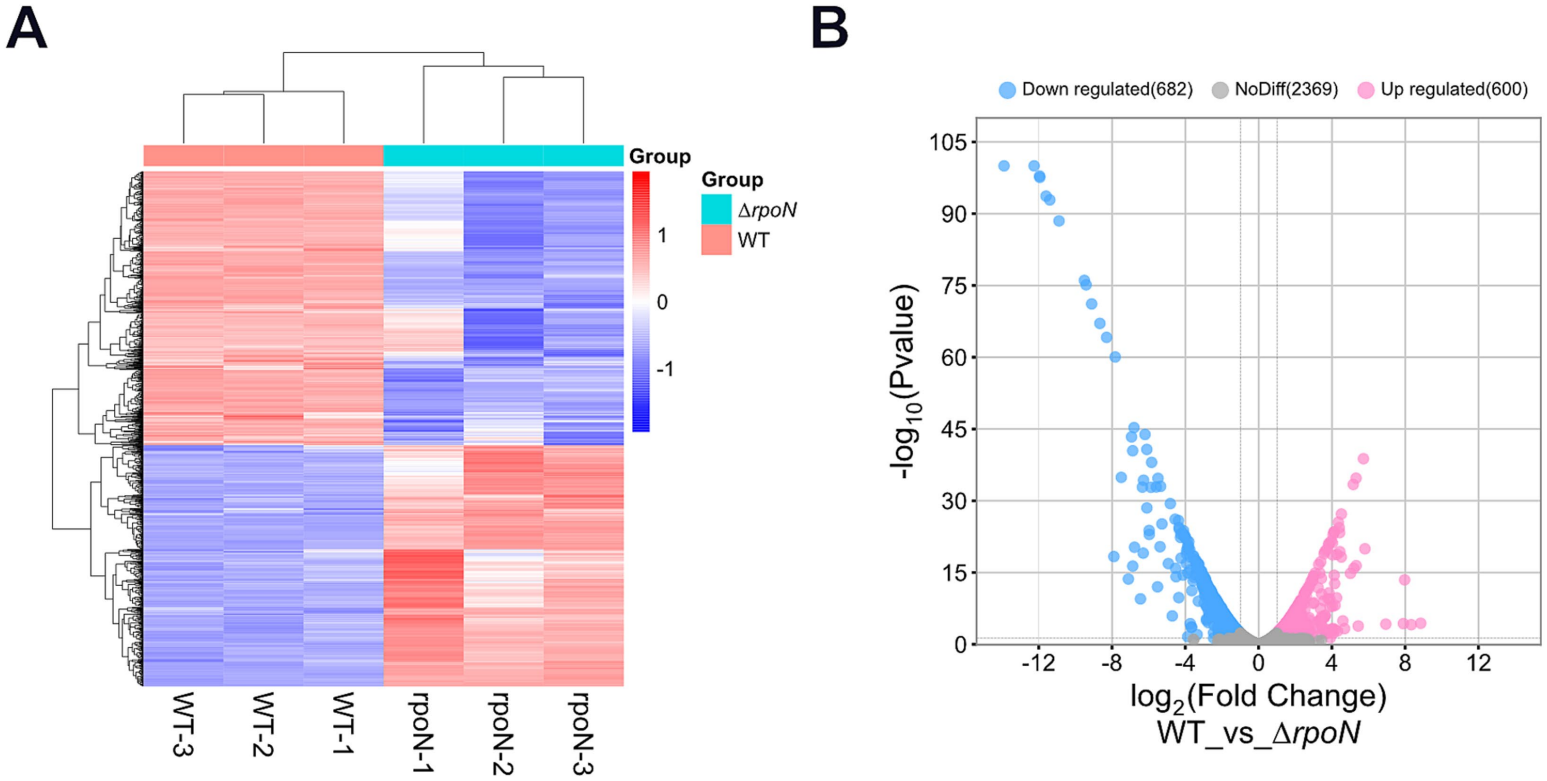
Transcriptome analysis. **(A)** Hierarchical clustering analysis of differentially expressed genes, with genes represented horizontally and samples vertically, each column representing a single sample; red indicates high-expression genes, and blue indicates low-expression genes; genes with high expression correlation are grouped together; **(B)** Volcano plot of differentially expressed genes, with the horizontal axis representing log2FoldChange and the vertical axis representing −log10(*p*-value). Blue dots on the left indicate downregulated genes in this group (682), pink dots on the right indicate upregulated genes (600), and grey dots represent genes with non-significant differential expression (2369).

To further investigate transcriptional changes in the Δ*rpoN* mutant compared to the WT strain, we performed comparative transcriptomic analyses during logarithmic growth. Overall, 600 genes were upregulated, 682 were downregulated, and 2,369 showed no significant change in expression (Figures 6A,B). As expected, expression of the *rpoN* gene was nearly undetectable.

The top 10 upregulated genes in the Δ*rpoN* mutant revealed significant enrichment in genes related to phosphate transport and transcriptional regulation. The phosphate transporter system (Pts), the transcription levels of phosphate ABC transporter permease (PstA), phosphate ABC transporter permease subunit (PtsC), phosphate ABC transporter ATP-binding protein (PstB), phosphate signaling complex protein (PhoU), and PTS sugar transporter subunit IIA were upregulated by 460, 322, 239, 123, and 56 times, respectively (Table 1). Factors related to transcriptional regulation, such as the helix-turn-helix transcriptional regulators (CD22140, CD22150) and the PRD domain-containing protein, were upregulated by 250, 40, and 43 times, respectively. Additionally, the hypothetical protein (CD02921) showed a transition from non-transcriptional to low-level transcription, and cytosine permease was upregulated by 53 times.

**Table 1:** Top 10 genes with expression changes in WT and ∆*rpoN* strains.

ID	Gnee_ID	Name	Average expression level		Fold change	Up/Down
			WT	∆*rpoN*		
1	CD02921	Hypothetical protein	0	6.58	∞	Up
2	CD32620	Phosphate ABC transporter permease PstA	10.81	4980.43	460.30	Up
3	CD32630	Phosphate ABC transporter permease subunit PstC	18.68	6008.77	321.60	Up
4	CD22140	Helix-turn-helix transcriptional regulator	23.95	5998.25	250.37	Up
5	CD32610	Phosphate ABC transporter ATP-binding protein PstB	16.13	3858.21	239.10	Up
6	CD32600	Phosphate signaling complex protein PhoU	18.25	2245.55	122.99	Up
7	CD02060	PTS sugar transporter subunit IIA	2.18	122.20	55.87	Up
8	CD27380	Cytosine permease	369.68	19509.81	52.77	Up
9	CD25110	PRD domain-containing protein	235.03	10169.41	43.26	Up
10	CD22150	Helix-turn-helix transcriptional regulator	2.21	88.81	40.02	Up
11	CD03950	Isocaprenoyl-CoA:2-hydroxyisocaproateCoA-transferase HadA	80512.66	5.28	15236.54	Down
12	CD04010	Electron transfer flavoprotein subunit alpha/FixB family protein	93134.65	19.04	4891.38	Down
13	CD04000	Electron transfer flavoprotein subunit beta/FixA family protein	70809.43	17.83	3970.93	Down
14	CD03990	Acyl-CoA dehydrogenase	65566.85	16.64	3940.12	Down
15	CD03960	2-hydroxyisocaproyl-CoA dehydratase activator HadI	48747.54	15.69	3106.41	Down
16	CD03980	(R)-2-hydroxyisocaproyl-CoA dehydratase subunit beta	59699.45	22.13	2696.17	Down
17	CD03970	(R)-2-hydroxyisocaproyl-CoA dehydratase subunit HadB	50590.77	26.56	1904.21	Down
18	CD32370	Proline racemase	73858.60	101.27	729.29	Down
19	CD32410	D-proline reductase (dithiol) protein PrdB	60239.28	88.26	682.48	Down
20	CD32360	TSUP family transporter	26659.23	48.14	553.71	Down

Conversely, the top 10 downregulated genes were largely associated with isovalerate metabolism and redox reactions. Genes encoding enzymes in the isovalerate pathway, such as isocaprenoyl-CoA-2-hydroxyisocaproate CoA-transferase (*hadA*), 2-hydroxyisocaproyl-CoA dehydratase activator (*hadI*), and subunits of (R)-2-hydroxyisocaproyl-CoA dehydratase (*hadB*), were downregulated by 15,237, 3,106, and 2,696 times, respectively. Electron transfer proteins, including electron transfer flavoprotein subunit alpha/FixB and beta/FixA family protein, were downregulated by 4,891 and 3,971 times, respectively. Acyl-CoA dehydrogenase was downregulated by 3,940 times. Proline racemase and D-proline reductase (dithiol) protein (PrdB) were downregulated by 729 and 682 times, respectively, and the TSUP family transporter by 554 times (Table 1).

### 3.7 Role of RpoN in virulence in a hamster model of CDI

**Figure 7:**
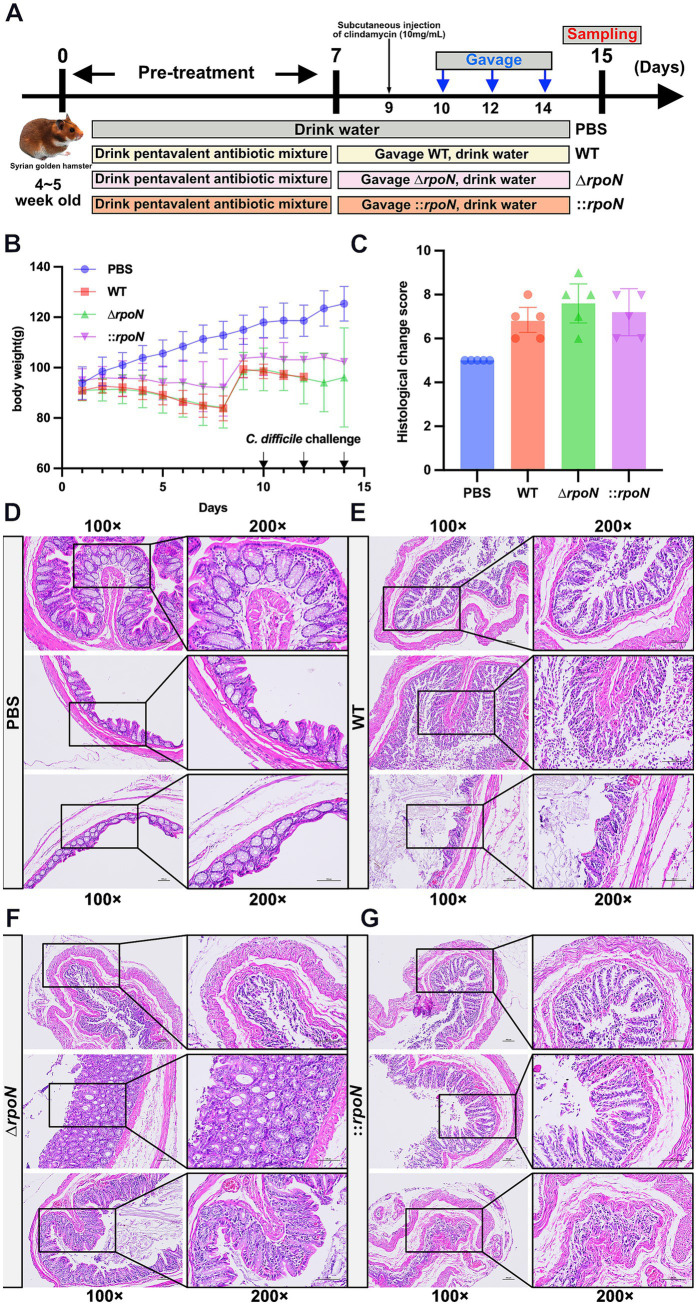
Construction of CDI animal model and the pathology of WT, ∆*rpoN*, and ⸬*rpoN* strains. **(A)** Schematic diagram of the construction process of CDI golden hamster model; **(B)** Daily weight change records of mice; **(C)** The H&E histopathological scores for the colon of the PBS, WT, Δ*rpoN*, and ⸬*rpoN* treated groups; **(D–G)** The H&E staining results for the PBS, WT, Δ*rpoN*, and ⸬*rpoN* groups. Data are expressed as mean ± SEM (*n* = 5 per group).

To further evaluate the pathogenicity of the WT, ∆*rpoN*, and ⸬*rpoN* strains, we utilized a hamster infection model and assessed disease progression through phenotypic observations and colonic histopathology. Compared to the control (PBS) group, all experimental groups (WT, ∆*rpoN*, and ⸬*rpoN*) exhibited diarrhea, characterized by soft stool and wet tail. Concurrent with continuous antibiotic administration for 7 days (Chen et al., 2008; Geeraerts et al., 2015; Mooyottu et al., 2017), body weight gradually decreased in all infected groups. Following antibiotic discontinuation (days 8 to 10), body weight partially recovered in the experimental groups (Figures 7A,B).

Histopathological analysis revealed differences in colonic tissue damage among the groups (Figure 7C). The control (PBS) group exhibited intact colonic morphology with clear layer structure, abundant glands, and regular arrangement of mucosal epithelial cells. Minimal damage was observed, with a few epithelial cells showing shrinkage and cytoplasmic staining, and a small number of inflammatory cells in the interstitium (Figure 7D). In the WT group, the colonic tissue retained clear layer structure, but mucosal epithelial cells were disorganized, with damage, detachment, and disintegration observed. Mild atrophy and loose arrangement of muscle layer cells were also noted, along with a few inflammatory cells (Figure 7E). The ∆*rpoN* group displayed the most severe colonic damage, characterized by loss of mucosal cells, disordered epithelial arrangement, extensive damage and disintegration of epithelial cells, sparse glands, incomplete or missing crypts, and obvious atrophy and sparse distribution of muscle layer cells. A large number of inflammatory cells were present in the interstitium (Figure 7F). Compared to the WT group, the ∆*rpoN* group showed increased crypt loss, epithelial surface damage and shedding, reduced mucus secretion, ulceration, and immune cell infiltration (Figures 7E,F). The ⸬*rpoN* group exhibited histopathology similar to the WT group, with clear layer structure, disorganized epithelial cells, some damage, detachment, and shrunken cell bodies, mild muscle layer atrophy, and a few inflammatory cells (Figure 7G). These findings suggest that the ∆*rpoN* strain exhibits increased pathogenicity in hamsters, correlating with the observed increase in toxin expression.

## 4 Discussion

The σ^54^ factor, essential for transcription in bacteria, forms a complex with RNA polymerase, enabling stable binding to promoter regions and initiating gene expression (Buck and Cannon, 1992). Consequently, σ^54^-dependent transcription factors exert broad regulatory effects on microbial physiology, influencing processes like transcription, carbon and nitrogen metabolism (Débarbouillé et al., 1991; Peng et al., 2015; Keffeler et al., 2021), toxin production, sporulation (Clark et al., 2022), motility (Douillard et al., 2009), biofilm formation (Da Silva Neto et al., 2008; Yang et al., 2020), secretion systems, and antibiotic synthesis (Liu et al., 2018). To investigate the role of σ^54^ in *C. difficile*, we generated an in-frame deletion mutant (∆*rpoN*) of the CD630 strain. Phenotypic analysis of this mutant revealed significant alterations, including reduced motility, autolysis rate, extracellular granule number, antibiotic resistance, H_2_S production, and glucose utilization. Notably, the ∆*rpoN* mutant exhibited significantly increased expression of the toxin genes *tcdA* and *tcdB*.

The increased expression of toxin genes in the ∆*rpoN* mutant aligns with previous observations in other *C. difficile* strains (Dubois et al., 2016), including BI-1 and CDC1 (Clark et al., 2022), and may result from competition between RpoN and the toxin-specific sigma factor TcdR for RNA polymerase, a mechanism consistent with the role of SigH (Saujet et al., 2011). Furthermore, upregulation of the AgrD1(CD27491)/AgrB1(CD27500) two-component system, approximately 2.78-fold and 2.29-fold respectively, likely contributes to the elevated expression of *tcdA* and *tcdB* (Ahmed et al., 2020). As expected, the increased production of TcdA and TcdB conferred enhanced virulence in a Vero cell model, and this increased pathogenicity was also confirmed in golden hamsters, suggesting that toxin gene expression is a primary driver of virulence. While other factors, such as adherence to intestinal epithelial cells and carbon source uptake, can also influence *C. difficile* pathogenicity (Janoir, 2016), our findings highlight the critical role of RpoN in regulating toxin production and subsequent virulence.

The ∆*rpoN* mutant of the CD630 strain also exhibited significantly increased autolysis rates, similar to observations in the CDC1 strain. However, unlike the BI-1 strain (Clark et al., 2022), it did not display cell aggregation. Interestingly, we found that the number of granules attached to the surface of the Δ*rpoN* strain was significantly reduced. Polyphosphate is widely distributed in both abiotic environments and living organisms. During microbial growth, phosphate is often actively acquired from external media and polymerized into longer-chain polymers via enzymatic reactions, serving as an intracellular storage depot. The formation of polyphosphate granules is intimately coupled to bacterial metabolic processes. Literature indicates that PG can bolster cell survival under stressful conditions, promote bacterial endospore formation (Lyratzakis et al., 2024), facilitate biofilm development (Zhang et al., 2005), and maintain outer membrane structure—for instance, a *Pseudomonas aeruginosa* PAO1 mutant deficient in *ppk1* exhibits disrupted exopolymers and malformed outer membrane structure (Li et al., 2007). Therefore, the reduction in granule formation may lead to decreased stress resistance in *C. difficile*, which is consistent with our findings.

We observed significant upregulation of two autolysin genes in the ∆*rpoN* mutant: cell wall protein 19 (*cwp19*) and autolysin (*acd*). Cwp19 encodes a peptidoglycan-degrading enzyme with lytic transglycosylase activity (Wydau-Dematteis et al., 2018), while *acd* is an N-acetylglucosaminidase that hydrolyzes peptidoglycan bonds (Dhalluin et al., 2005). The increased expression of these autolysins likely contributes to the observed autolysis and may facilitate the release of toxins into the environment, potentially explaining the enhanced virulence observed in cell and animal models.

Consistent with previous reports demonstrating a role for *rpoN* in flagellum assembly regulation in other microorganisms (e.g., Liu et al., 2021, in *Pseudomonas fluorescens*), our KEGG analysis revealed a significant enrichment of flagellar assembly genes among downregulated cellular processes. Specifically, expression of genes encoding proteins involved in flagellum assembly, including *fliL/M/N/Z/P/Q/R* (CD02580/CD02700/CD02710/CD02590/CD02600/CD02610/CD02620), *flhA/B* (CD02630/CD02620), *flgG* (CD02680 and CD02690), *fliC* (CD02390), and *fliA* (CD02660), was significantly reduced. These genes encompass structural components, regulatory factors, and chemotaxis genes, suggesting that RpoN positively regulates flagellar assembly. Consequently, the reduced motility observed in the ∆*rpoN* mutant of *C. difficile* is directly linked to the downregulation of these flagellar genes.

Previous research has demonstrated that deletion of the *rpoN* gene can alter antibiotic susceptibility in various pathogenic bacteria. For instance, *Pseudomonas fluorescens* ∆*rpoN* mutants exhibit increased sensitivity to nine antibiotics (Liu et al., 2021), while a clinical *Pseudomonas aeruginosa* strain with a deleted *rpoN* gene shows increased susceptibility to several beta-lactams (Lloyd et al., 2019). Similarly, *Borrelea burgdoeferi* mutants with *rpoN* mutations display increased sensitivity to doxycycline (Sapi et al., 2016). However, the impact of *rpoN* deletion on antibiotic susceptibility is strain-dependent; in *Pseudomonas aeruginosa*, a ∆*rpoN* mutant in the logarithmic growth phase showed increased resistance to quinolones and carbapenems, linked to upregulation of pyoverdine expression (Viducic et al., 2007). In our study, the CD630 ∆*rpoN* mutant exhibited increased susceptibility to all seven antibiotics tested: *β*-lactams (amoxicillin, ampicillin, cefoxitin), nitroimidazoles (metronidazole), glycopeptides (vancomycin), fluoroquinolones (norfloxacin), and aminoglycosides (kanamycin). This increased susceptibility may be attributed to two primary mechanisms: (1) downregulation of antibiotic resistance genes, such as a 4.43-fold decrease in the expression of the vancomycin resistance gene VanT (CD16280, part of the VanC cluster, which influences vancomycin resistance), and (2) disruption of intracellular antioxidant mechanisms, evidenced by reduced synthesis of thiol compounds (CD00360, CD01180, CD07280, CD19170, CD20340, CD23800, CD23810, CD31740 oxidoreductase activity), potentially hindering the clearance of reactive oxygen species (ROS) generated under antibiotic (Albesa et al., 2004; Van Acker and Coenye, 2017). These findings suggest that RpoN represents a potential target for combination therapy in *C. difficile* infections.

Our transcriptomic analysis comparing WT and ∆*rpoN* mutants revealed significant downregulation of genes involved in glucose metabolism. Specifically, we observed a 6.69-fold and 7.68-fold decrease in expression of the sugar PTS system EIIA component (*crr*), and a 3.70-fold, 4.92-fold, 2.02-fold, 3.41-fold, 2.03-fold, 2.33-fold, 2.86-fold, 2.29-fold, and 2.01-fold reduction in *ALDO* (fructose-bisphosphate aldolase, class I), *GAPDH* (glyceraldehyde-3-phosphate dehydrogenase), *pgk* (phosphoglycerate kinase), *pyk* (pyruvate kinase), *aceE* (pyruvate dehydrogenase E1 component), *dlat* (pyruvate dehydrogenase E2 component), *dld* (dihydrolipoyl dehydrogenase), and *adh* (alcohol dehydrogenase), respectively. This widespread downregulation likely contributes to the significantly reduced glucose utilization observed in the ∆*rpoN* mutant, suggesting that RpoN positively regulates glucose metabolism. Unlike observations in Gram-positive bacteria, regulation of nitrogen metabolism by RpoN was less prominent in *C. difficile*. We detected only modest upregulation of *hcp* (2.53-fold) and *glnA* (2.47-fold). Regarding cysteine metabolism, expression of *cysK* (cysteine synthase) and *cysE* (serine O-acetyltransferase) was dramatically reduced (68.68-fold and 57.33-fold, respectively) in the ∆*rpoN* mutant, leading to decreased L-cysteine synthesis. Given that L-cysteine can be converted to pyruvate and hydrogen sulfide (H_2_S), the observed reduction in RpoN-mediated cysteine synthesis may explain the decreased H_2_S production.

In conclusion, we constructed and characterized an in-frame deletion mutant of the *rpoN* gene in *C. difficile* through phenotypic analysis and RNA-Seq. Our results demonstrate that RpoN significantly impacts *C. difficile* motility, antibiotic susceptibility, toxin production, and virulence by regulating the expression of numerous genes. These findings establish RpoN as a global regulator in *C. difficile* and suggest it as a promising therapeutic target for reducing virulence and antibiotic resistance.

## Data Availability

The RNA-seq raw data (RNA-seq of *Clostridioides difficile* Δ*rpoN* mutant against wild-type control) was deposited in the ArrayExpress database (https://www.ebi.ac.uk/biostudies/arrayexpress) under the accession number of E-MTAB-14512.
