# Supplementary material for: The sigma factor σ54 (rpoN) functions as a global regulator of antibiotic resistance, motility, metabolism, and virulence in Clostridioides difficile
Source: Front Microbiol. 2025 Apr 29;16:1569627. doi: 10.3389/fmicb.2025.1569627 (PMC12069270; doi:10.3389/fmicb.2025.1569627)
Supplement: Supplementary file 1 [file Data_Sheet_1.docx]

Supplementary Material

# Supplementary Figures and Tables

## Figure S1 Gene context and gene deletion workflow

The *rpoN* (CD31760) gene is located in the RNA polymerase factor Sigma-54 (σ^54^) cluster, flanked by CD31750 and CD31770 genes, which encoded transcriptional regulator SorC family (*cggR*) and selenium-dependent xanthine dehydrogenase (*xdh*), respectively. The *rpoN* gene contains three domains, from the N-terminus to the C-terminus are the activator interacting domain, the core binding domain, and the DNA binding domain (Figure S1). The technical workflow for the *rpoN* gene knockout is provided in Figure S1B to E, and the molecular validation data confirming the successful deletion are presented in Figure S2.

**Figure S1. Gene context of the *rpoN* gene and CRISPR-Cpf mediated gene deletion of the *rpoN*.**

(A) Gene context of the *rpoN* gene and its primary and tertiary structure, the *rpoN* gene is flanked by CD630_31750 and CD630_31770 genes; (B) Construction of mother vector pYJY3-S, the negative selection marker (*pyrF'*), which originates from the *Clostridium beijerinckii* NCIMB 8052 strain, was added to the pWH34 plasmid to obtain the plasmid pYJY3-S; (C) Construction of the *rpoN* gene knockout plasmid pYJY3. The pre-crRNA expression cassette PsRNA-pre-crRNA, upstream homology arm (Up-arm), and downstream homology arm (Down-arm) were assembled into the *Btg*ZI site of pYJY3-S in a single step using One-Step Cloning kit; (D) The assemble of the gene-targeting effector. AsCpf1 nucleases process pre-crRNA to mature crRNA and assemble the gene-targeting effector; (E) The workflow of generating ∆*rpoN* mutant and curing the gene-targeting plasmid. Step 1, Plasmid pYJY3 was conjugated into *C. difficile* under the selection of Tm; Step 2, lactose was added to the BHIS medium to induce the expression of the AsCpf1; Step 3, the pYJY3 in the ∆*rpoN* deletion mutant was cured by counter-selection on CDMM+5-FOA medium.

## Figure S2 Plasmid construction and determination of phenotypes of mutant strains

The pYJY3 plasmid was conjugated into *C. difficile* 630 strain. Twenty-three colonies were randomly selected from the transformants and screened for the *rpoN* gene deletion mutant (∆*rpoN*) using primers HW557/HW558 (Table S2). The results showed that all selected colonies were ∆*rpoN* mutants, with a efficiency of 100% (1,673 bp vs. 493 bp) (Figure S2A). The ∆*rpoN* transformants were then streaked onto BHIS medium containing 5-FOA to cure the gene-targeting plasmid pYJY3. Further sequencing results indicated that a 1,180 bp gene deletion was successfully achieved in the plasmid-cured ∆*rpoN* mutant strain (Figure S2B). The pYJY3 plasmid was transformed into the wild-type *C. difficile* 630 (WT) using the same conjugation and screening methods to obtain the complemented strain (::*rpoN*).

**Figure S2. Screening of the ∆*rpoN* mutant strain.**

(A) Diagnostic PCR was performed to verify gene deletion of the *rpoN* gene using primer pair HW557/HW558. The WT and ∆*rpoN* mutants produce 1,673 bp and 493 bp PCR amplicon, respectively; (B) Gene sequencing confirmed that 1,180 bp of DNA sequence within the *rpoN* gene was deleted from *C. difficile* 630 genome.

## Figure S3 Construction of different gene mutants

## The workflow for generating the Δ*rpoN* mutant, curing the gene-targeting plasmid, and constructing the ::*rpoN* complementary mutant is shown in Figure S3.


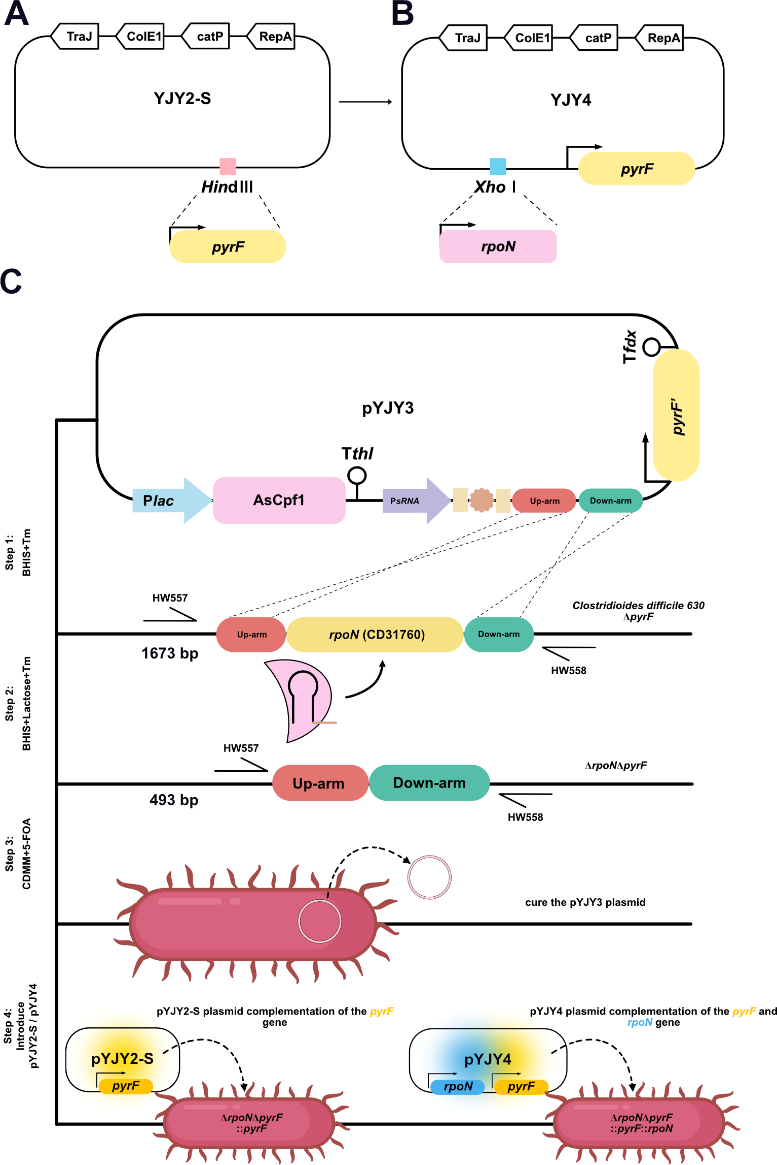


**Figure S3.** The workflow of generating *∆rpoN* mutant, curing the gene-targeting plasmid and construction of ::*rpoN* complementary mutant.

(A) The *pyrF* gene fragment was assembled with the *Hin*dIII-linearized pMTL82151 plasmid to produce pYJY2-S; (B) The *rpoN* gene fragment was assembled with the *Xho*I-linearized pYJY2-S plasmid to obtain pYJY4; (C) Step 1, Plasmid pYJY3 was conjugated into *C. difficile* under the selection of Tm; Step 2, lactose was added to the BHIS medium to induce the expression of the AsCpf1; Step 3, the pYJY3 in the *∆rpoN* deletion mutant was cured by counter-selection on CDMM+5-FOA medium; Step 4, introduce pYJY2-S and pYJY4 into the Δ*rpoN*∆*pyrF* mutant strain, resulting in complemented strains Δ*rpoN*∆*pyrF*::*pyrF* and Δ*rpoN*∆*pyrF*::*pyrF*::*rpoN.*

## Figure S4 The *rpoN* gene impacts carbon source utilization

Growth rates of WT, Δ*rpoN*, and ::rpoN strains utilizing various carbon sources were assessed (Figure S4). While growth on mannitol, mannose, fructose, and glucose was observed for all strains, the Δ*rpoN* mutant exhibited significantly reduced growth rates compared to the WT strain, particularly in glucose and mannitol media. Notably, complementation of the Δ*rpoN* mutation with ::*rpoN* restored growth rates to WT levels. These findings suggest that the *rpoN* gene plays a role in regulating glucose uptake efficiency.


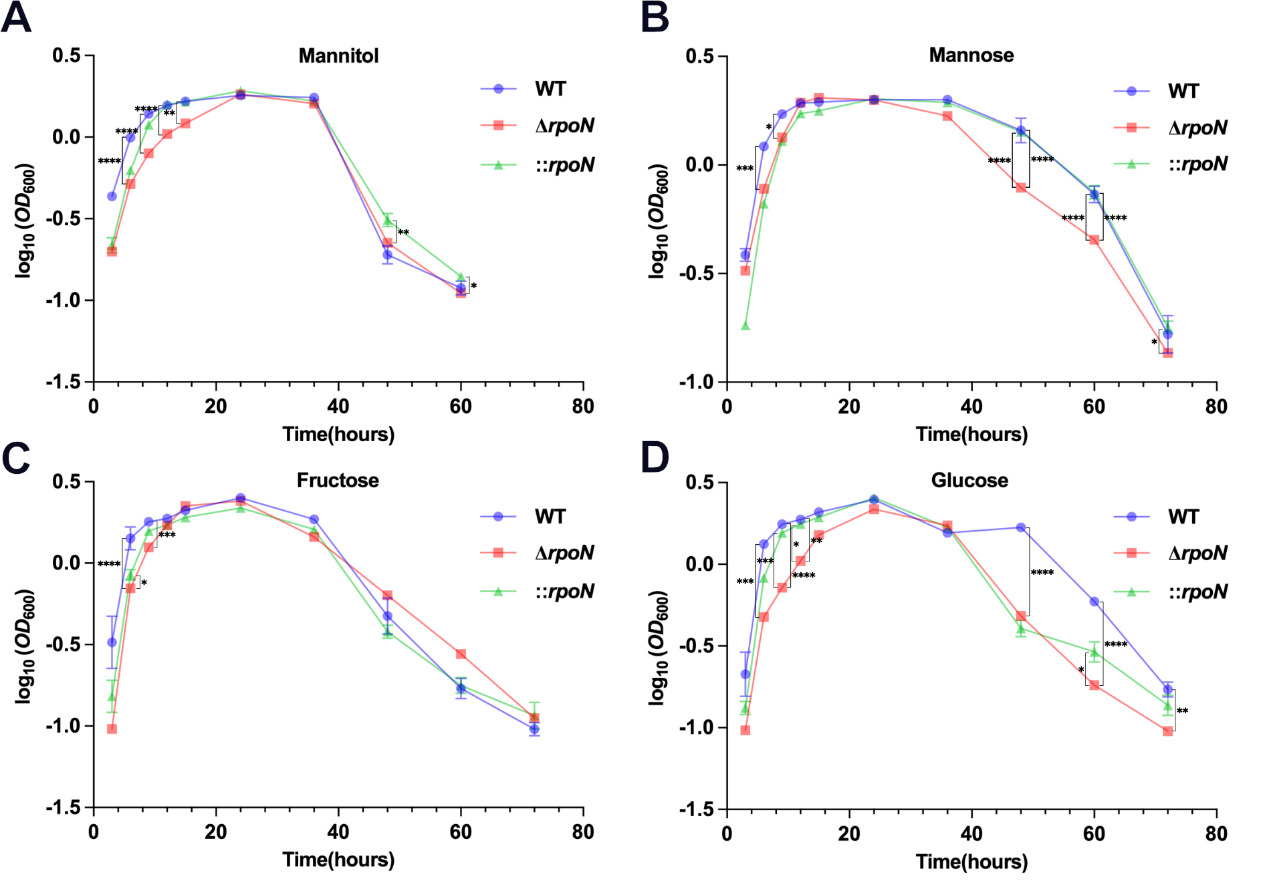


**Figure S4 Sole carbon source utilization by WT, ∆*rpoN*, and ::*rpoN* strains.**

# The vertical coordinate is the value of *OD*_600_, and the horizontal coordinate is the incubation time (hours). Growth curve of the WT, ∆*rpoN* and ::*rpoN* strains in the CDMM medium supplemented with mannitol (A), mannose (B), Fructose (C), or Glucose (D) as the sole carbon source. Data are presented as mean ± SEM. Statistical significance was determined using two-way ANOVA, followed by Tukey's post hoc test. * P≤0.05, ** P≤0.01, *** P≤0.001, **** P≤0.0001.

## Table S1 Bacterial and plasmids used in this study

| **Strains or Plasmids** | **Relevant characteristics** | **Reference or source** | |
| --- | --- | --- | --- |
| **Strains** |  | |  |
| ***E. coli*** |  | |  |
| NEB Express Competent *E. coli*  (High efficiency) | *fhuA2 [Ion] ompT gal sulA11 R(mcr-73::miniTn10--Tet^S^)2[dcm]R(zgb-210::Tn10--Tet^S^)endA delta(mcrC-mrr)114::IS10* | | NEB |
| *E. coli* CA434 | *hsd20(r^B^-, m^B^-), recA13, rpsL20, leu, proA2, with IncPb conjugative plasmid R702* | | (1) |
|  |  | |  |
| ***Clostridioides difficile*** |  | |  |
| *C. difficile* 630 | WT stain | | ATCC |
|  |  | |  |
| **Plasmids** |  | |  |
| pMTL82151 | pBP1 ori, Cm^R^, ColE1 ori, TraJ, *lacZ* α fragment | | (2) |
| pWH37 | pBP1 ori, ColE1 ori, TraJ, lacZ α fragment, sRNAP:: crRNA | | (3) |
| pWH34 | Derived from pMTL82151, *E. coli*-*C. difficile* shuttle vector, iLacP::AsCpf1, BtgZI-BtgZI double sites, “Chassis” plasmid for gene-targeting plasmid construction, Cm^R^/Tm^R^ | | (3) |
| pYJY2-S | Derived from pMTL82151, for complement for *pyrF* gene, containing *pyrF* gene and promoter | | This work |
| pYJY3-S | Derived from pWH34, including *pyrF`* gene of *Clostridium* *beijerinckii* NCIMB 8052 | | This work |
| pYJY3 | Deletion of *rpoN* gene, including pre-crRNA expression cassette PsRNA-pre-crRNA，upstream and downstream homologous arms of *rpoN*, derived from pYJY3-S | | This work |
| pYJY4 | Derived from pYJY2-S, for complement for *rpoN* gene, containing *rpoN* gene and promoter | | This work |

## Table S2 Primers used in this study

| **Primers** | **Sequences(5’-3’)** |
| --- | --- |
| HW520 | aaagttaaaagaagaaaatagaaatATAATCTTTAATTTGAAAAGATTTA |
| HW521 | ttctcagtctcaatggcgctactATCTACAAGAGTAGAAATTA |
| HW523 | gaaacagctatgaccGCGGCCgtcaaatagaaggtggagtagt |
| HW524 | catcacttaaagcttctgagactgagttaat |
| HW525 | gtctcagaagctttaagtgatgaccatatatctaagtt |
| HW526 | gtatataagatacaGCGGCCtataccctacgtgttttcct |
| HW554 | GGGAGACTTGAGTGCAGGAG |
| HW555 | GTGCCTCAGCGTCAGTTACAGT |
| HW557 | tagcccaatctacattcatc |
| HW558 | tacagttcctactgcacca |
| HW625 | CGCGTccatggagatcagctTACACATGTGCCATGTCAG |
| HW626 | tgcatgtctgcaggcctcgaTTATATGTTCTTCACTGCTTCTC |
| HW684 | gtcacgcgtccatggagatcgcttggtactgttatgactcaag |
| HW685 | agcttgcatgtctgcaggccagcccaatctacattcatctc |
| HW693 | tgaaatggcatctagtacaagca |
| HW694 | tggcaactgtccttcttgca |
| HW731 | gcctgcagacatgcaagcatgcaaacagtgcaaaaaat |
| HW734 | acgacggccagtgccaagccctgggccattagataataag |
| HW885 | gagaagggcataatgagaacgg |
| HW886 | tgcatgacaccatcttcacc |
| HW887 | agctttcgctttaggcagtg |
| HW888 | atggctgggttaaggtgttg |

**Supplementary References**

1. Williams, D. R., Young, D. I., & Young, M. (1990). Conjugative plasmid transfer from *Escherichia coli* to *Clostridium acetobutylicum*. *Microbiology*, *136*(5), 819-826.

2. Heap, J. T., Pennington, O. J., Cartman, S. T., & Minton, N. P. (2009). A modular system for *Clostridium* shuttle plasmids. *Journal of microbiological methods*, *78*(1), 79–85.

3. Hong, W., Zhang, J., Cui, G., Wang, L., & Wang, Y. (2018). Multiplexed CRISPR-Cpf1-Mediated Genome Editing in *Clostridium difficile* toward the Understanding of Pathogenesis of *C. difficile* Infection. *ACS synthetic biology*, *7*(6), 1588–1600.
